# Comparison between Single-Dose and Two-Dose Psilocybin Administration in the Treatment of Major Depression: A Systematic Review and Meta-Analysis of Current Clinical Trials

**DOI:** 10.3390/brainsci14080829

**Published:** 2024-08-18

**Authors:** Gianmarco Salvetti, Daniele Saccenti, Andrea Stefano Moro, Jacopo Lamanna, Mattia Ferro

**Affiliations:** 1 Department of Psychology, Sigmund Freud University of Milan, 20143 Milan, Italysaccenti.phd@milano-sfu.it (D.S.); m.ferro@milano-sfu.it (M.F.); 2 Brain and Behaviour SFU Lab, Sigmund Freud University of Milan, 20143 Milan, Italy; 3 Center for Behavioral Neuroscience and Communication (BNC), Vita-Salute San Raffaele University, 20132 Milan, Italy; 4 Faculty of Psychology, Vita-Salute San Raffaele University, 20132 Milan, Italy

**Keywords:** major depressive disorder, MDD, treatment-resistant depression, TRD, psilocybin, psychedelics

## Abstract

Current pharmacological treatments for major depressive disorder (MDD) are often only partially effective, with many patients experiencing no significant benefit, leading to treatment-resistant depression (TRD). Psilocybin, a classical serotonergic psychedelic, has emerged as a notable emerging treatment for such disorders. The aim of this systematic review and meta-analysis is to summarize and discuss the most recent evidence about the therapeutic effects of single-dose and two-dose psilocybin administration on the severity of depressive symptoms, as well as compare the efficacy of these interventions among patients with a primary diagnosis of MDD or TRD. Articles were collected from EBSCOhost and PubMed following the PRISMA guidelines, yielding 425 articles with 138 duplicates. After screening 287 records, 12 studies met the eligibility criteria and were included in the review. A quantitative analysis of the studies indicates that psilocybin is highly effective in reducing depressive symptoms severity among patients with primary MDD or TRD. Both single-dose and two-dose psilocybin treatments significantly reduced depressive symptoms severity, with two-dose administration sometimes yielding more pronounced and lasting effects. However, it is unclear if this was solely due to dosage or other factors. Future research should include standardized trials comparing these dosing strategies to better inform clinical practice.

## 1. Introduction

Major depressive disorder (MDD) is one of the most diagnosed psychiatric disorders worldwide, and it afflicts between 4% and 5% of the global population each year [1,2]. In addition to being extremely disabling in most cases, with symptoms such as significant mood swings, anhedonia, and recurrent thoughts about death, it also affects the individual’s vital spheres of appetite, sleep, psychomotor activity, and attention [3]. MDD comes with a heavy financial burden as well [4]. During the eight-year period 2010–2018, the economic burden associated with adults with MDD in the United States grew from $236.6 billion to $326.2 billion, an increase of 37.9% [5]. Currently, the management of MDD involves the use of various psychotherapeutic approaches, such as cognitive-behavioral therapy (CBT) and interpersonal therapy (IPT), as well as assorted pharmacological interventions, including selective serotonin reuptake inhibitors (SSRIs), tricyclic antidepressants (TCAs), as well as serotonin and norepinephrine reuptake inhibitors (SNRIs) [6]. However, despite the availability of such treatments, a significant percentage of patients, around 30%, do not respond positively to two or more of the psychotherapeutic or pharmacological strategies available on the market [7]. In these cases, the label of treatment-resistant depression (TRD) is given [8]. The drugs currently used for the treatment of MDD, other than being often ineffective [9], also present numerous side effects. The most frequently discussed aftereffects in the literature are: sexual problems and dysfunctions (that may persist even after stopping the treatment) [10,11], nausea [12], weight gain [13,14], and alterations to sleep structure (e.g., a prolongation of the REM phase and an increase in the number of awakenings) [12]. Furthermore, discontinuing SSRIs after prolonged use can result in withdrawal symptoms such as headaches, anxiety, agitation, and lethargy, although these tend to resolve within three weeks [15]. The latency period of these drugs is quite long, ranging from 2 to 12 weeks [16]. This latency could lead to adverse consequences, such as non-adherence to therapy and an increased risk of suicide [17]. Six meta-analyses on the suicide rate [18,19,20,21,22,23] discovered that individuals with MDD did not see a decrease in suicide ideation after SSRI therapy. Notably, a meta-analysis found that it was even higher [18].

Every important aspect that has been brought up emphasizes how crucial it is to look for novel, potentially effective, pharmaceutical treatments. Currently, among the new drugs explored for the treatment of MDD is psilocybin, i.e., a compound found within more than a hundred species of fungi, mostly belonging to the family *Psilocybe*. Psilocybin can be considered a psychotropic substance due to the hallucinogenic properties of its active metabolite, i.e., psilocin. In particular, psilocybin belongs to the category of classical serotoninergic psychedelics, and it is characterized by inducing altered states of consciousness through heterogeneous molecular mechanisms [24]. Indeed, psilocybin was originally thought to act as a serotonergic (5-HT) receptor agonist [25], specifically the 5HT-2A receptor, believed to be responsible for the substance’s potent hallucinogenic effects [26]. However, more recent investigations indicated that psilocin binds with high affinity to tropomyosin receptor kinase B (TrkB) receptors for brain-derived neurotrophic factor (BDNF), and its effects on synaptic plasticity as well as antidepressant-like behavior in mice depended on TrkB-BDNF signaling but were independent of 5-HT2A receptor activation [27].

In contrast to conventional antidepressant drugs, psilocybin, like other psychoactive substances (e.g., ketamine; [28,29]), showed a rapid onset of action, with significant improvements in depressive symptoms severity observed as early as the first few days after treatment [30]. Although ketamine has already shown strong response rates among MDD patients [31], its therapeutic effects are closely associated with the development of tolerance, dependence, and the potential risk of abuse, as well as the sporadic occurrence of severe adverse events (e.g., dissociation and sedation) and withdrawal-related symptoms [32,33]. Conversely, psylocibin shows a significantly lower physical dependence potential, and its side effects, including headache, nausea, and dizziness, appear milder and rarer [34,35,36]; yet, it can still cause tolerance, and psychological dependence might occasionally develop [37,38]. Psilocybin tends to be administered orally, and its effects last from two to six hours [39]. Importantly, drug delivery is accompanied by preparatory and integrative psychological sessions, forming an indispensable part of the broad treatment protocols [40,41]. Psychological guarantees not only support during the psychedelic experience to guarantee a reduction of adverse effects [42], but also a reflection and elaboration of the experiences lived during such a timeframe, thus allowing the remission of symptoms to be maintained even in the long term [43,44].

An issue that can be found upon careful analysis of clinical trials conducted with psilocybin for the treatment of MDD is that there is currently no unified treatment. This might be linked to the relative novelty of the research field, and conducting trials with different designs potentially allows to infer which modalities of drug use are most effective. The data currently available on psilocybin are increasing drastically, and between clinical trials, numerous differences can be observed in terms of psychological support (e.g., duration of sessions, support offered, psychotherapeutic orientation), dosage (e.g., varies in a range from 10 mg to 30 mg), and number of administrations (e.g., one, two, or more than two). Although one systematic review measuring the efficacy of the different numbers of dosing sessions already exists [45], there is no recent systematic review and/or meta-analysis in the literature comparing the efficacy of different numbers of psilocybin administrations in a sample of patients with a primary diagnosis of MDD or TRD, also discussing the evidence brought to light by the latest clinical trials.

The aim of this systematic review is to (1) summarize and discuss the current evidence on the therapeutic effects of single-dose and two-dose psilocybin administration on MDD and TRD core symptoms, and to (2) compare the efficacy of these two approaches in reducing depressive symptoms severity among patients with a primary diagnosis of MDD or TRD.

## 2. Materials and Methods

The present systematic review and meta-analysis was carried out referring to the Preferred Reporting Items for Systematic Reviews and Meta-Analyses (PRISMA) guidelines [46], and it was successfully registered on the International Prospective Register of Systematic Reviews (PROSPERO; ID: CRD42024543828).

### 2.1. Information Sources and Research Strategies

A literature search was conducted to identify studies using one or two doses of psilocybin for the treatment of MDD. The search was carried out within two electronic databases: PubMed and EBSCOhost, which comprise records retrieved from PsycINFO, PsycARTICLES, PSYNDEX: Literature and Tests, MEDLINE, and ERIC. The search string used was as follows: “(“psilocybin” OR “psilocybin-assisted”) AND (“major depressive disorder” OR MDD OR “treatment-resistant depression” OR TRD OR “secondary depression” OR “depression”) AND ((clinical trial) OR ((randomized controlled trial) OR (RCT)) OR ((open trial) OR (open-label)))”. The two databases were last searched on 27 February 2024.

### 2.2. Eligibility Criteria

Below are the eligibility criteria drawn up on the basis of the PICOs model [47], which allowed the selection of the studies in this systematic review. To be included in this systematic review, studies needed to meet the following inclusion criteria:

- Language: only articles in English were selected. This is for uniformity with other systematic reviews in the literature.
- Population: as it was the aim of this systematic review to investigate the efficacy of psilocybin with a focus on MDD, it was considered necessary to select only the population with a primary diagnosis of MDD, including subjects with TRD, a label of MDD itself.
- Types of studies: only data from clinical trials conducted on humans were taken into account.
- Intervention: only those studies involving the administration of a single dose or two doses of psilocybin were included, regardless of the specific dosage and mode of administration.
- Outcome: only studies that assessed a possible decrease in the severity of depressive symptoms among participants using standardized instruments were included.
- Comparison: the presence of one or more control groups that were treated with active (e.g., niacin) or standard placebo (e.g., saline) was not mandatory for inclusion.

Studies were excluded from the systematic review in the following cases:

- Population: trials with participants who did not report a diagnosis of MDD or reported it as a secondary diagnosis to other psychiatric disorders or other conditions of organic nature (e.g., cancer) were excluded.
- Types of studies: clinical trials performed on animal models, case studies, and systematic reviews were excluded.
- Intervention: studies in which other psychedelics than psilocybin, e.g., mescaline or bufotenine, were administered to patients were excluded, as well as studies involving no or more than two doses of psilocybin.
- Outcome: trial studies that did not include scales measuring depressive symptoms or in which timepoints were missing were excluded.

### 2.3. Study Screening and Selection Process

Using Zotero (Version 6.0.31, https://www.zotero.org accessed on 1 March 2024), the first author eliminated duplicates after conducting searches in the electronic databases and finding pertinent studies. Both the primary and secondary authors conducted independent screenings of all the data based on the inclusion and exclusion criteria. Two steps made up the screening process: (1) authors checked to see if preprints, conference presentations, research protocols, or non-clinical trial articles were retrieved; and (2) specific inclusion criteria related to participants, intervention, outcome measure, and study design were applied for the assessment of the remaining manuscripts. The situations that were determined to be ambiguous were further examined with the help of other authors.

### 2.4. Data Extraction Process

Data were extracted from the full text of the articles. The data extracted for this review were as follows: research design, sample details (number of participants, % women, mean age, and standard deviation), measurements conducted (rating scales used to measure depressive symptoms), participants’ diagnoses (with assessment of severity), dosages used (number of doses and quantity in mg), treatment structure (including information on psychological support and group treatment conditions), and the results that emerged regarding depressive symptoms. The extraction work was carried out by the first author with the help of the others in cases of ambiguity.

### 2.5. Risk of Bias in Individual Studies

The risk of bias was assessed using the updated Cochrane Risk-of-Bias tool for randomized controlled trials (RoB 2; [48]) for articles that had a randomized design, whereas using the Risk of Bias in Non-randomized Studies—of Intervention (ROBINS-I; [49]) for the studies lacking a control group. Employing the RoB2, five domains were assessed: (1) bias arising from the randomization process; (2) risk of bias due to deviations from the intended interventions; (3) risk of bias due to missing outcome data; (4) risk of bias in measurement of the outcome; and (5) risk of bias in selection of the reported result. On the other hand, using the ROBINS-I, seven domains were assessed: (1) bias due to confounding; (2) bias in selection of participants into the study; (3) bias in classification of interventions; (4) bias due to deviations from intended interventions; (5) bias due to missing data; (6) bias in measurement of outcomes; and (7) bias in selection of the reported results.

### 2.6. Statistical Analysis

Two separate meta-analyses were conducted to evaluate the treatment effect on patients’ depressive symptoms severity. First, we examined the effect size linked to the active intervention alone, considering the standardized mean difference (SMD), i.e., Cohen’s *d*, between post-treatment and baseline scores within the active treatment groups. Second, we tested whether there was a significant treatment effect compared to control, thus considering the SMD between post-treatment scores obtained by active and control groups, where applicable. The formula SD = SEM × √*n* (*n* = sample size) was used for the conversion of standard errors of the mean (SEM) into standard deviations (SD). Both meta-analyses were carried out using random effects models due to the large heterogeneity in the estimates. Funnel plots were employed in order to visually assess heterogeneity and potential outliers. Cochran’s Q was used to assess heterogenicity in the effect size distribution, while the I^2^ index (95% confidence interval) was employed to evaluate heterogeneity resulting from effect size variability and sampling error. Heterogeneity levels (I^2^) were categorized as low at 25%, moderate at 50%, and high at 75% [50]. To examine publication bias, Egger’s [51] test was computed. The analyses were performed using R (Version 4.4.1, https://www.r-project.org accessed on 15 June 2024).

## 3. Results

**Figure 1:**
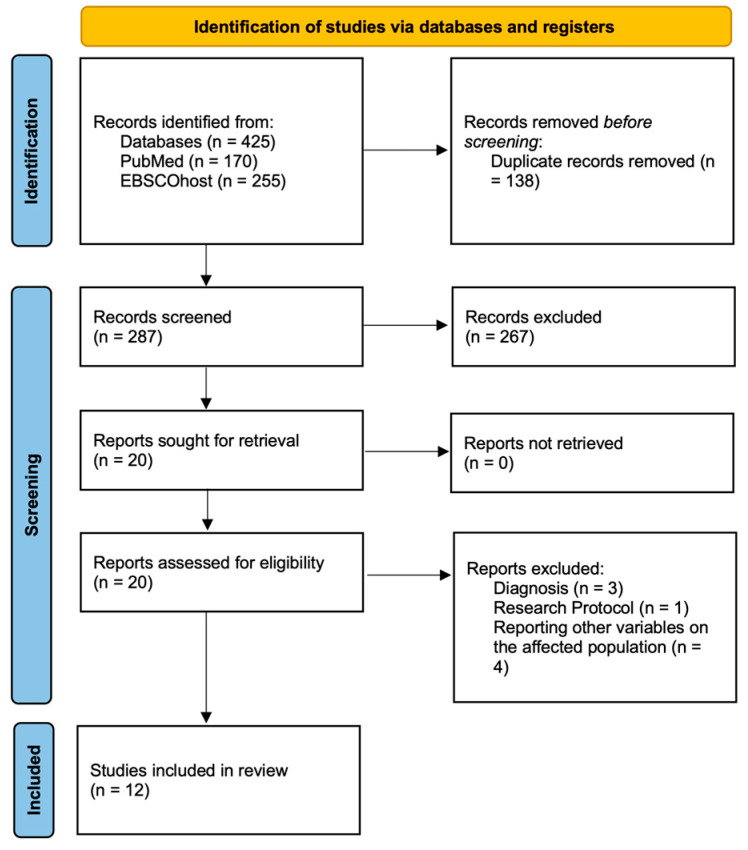
PRISMA Flow Chart. Adapted from Page et al. [46]. For more information, please visit: https://www.prisma-statement.org accessed on 1 April 2024.

The search results and the study selection process are depicted in the flowchart below (Figure 1). The search string, once entered into the two databases, produced a total of 425 results (of which 170 on PubMed and 255 on EBSCOhost). Following removal of duplicates (*N* = 138), 287 articles were screened. Following reading of the title and abstract 268 were excluded, leading to 20 studies. All studies were found; therefore, 20 trials were subjected to the final screening step by full text reading. A further 8 studies were excluded as they did not meet the eligibility criteria outlined above. Three studies were excluded because participants’ primary diagnosis was not MDD [52,53,54], one because it was not a clinical trial but a research protocol [40], and four because they reported other variables of no interest for the present review on the population affected [55,56,57,58]. Overall, the studies included in this review were 12 [30,59,60,61,62,63,64,65,66,67,68,69].

### 3.1. Risk of Bias Assessment

**Figure 2:**
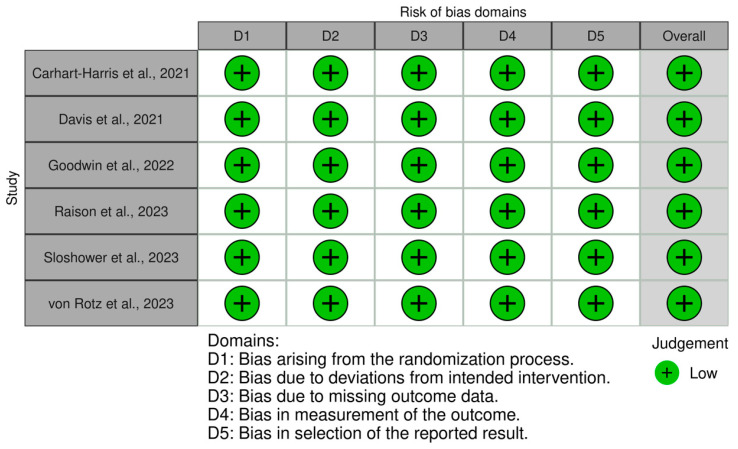
Traffic light plot showing risk of bias assessment of the included studies using the RoB2 tool [30,62,64,67,68,69].

Using the RoB2 tool, all six randomized controlled studies were judged to have a low risk of bias in all five domains, resulting in an overall low risk of bias (Figure 2).

**Figure 3:**
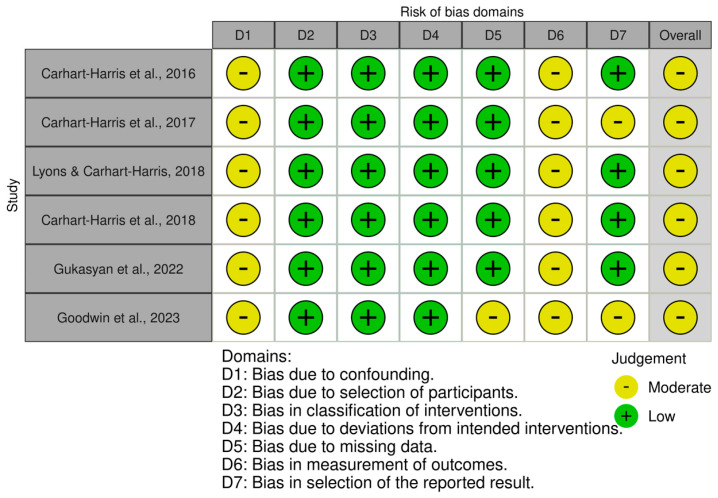
Traffic light plot showing the risk of bias assessment of the included studies using the ROBINS−I tool [59,60,61,63,65,66].

On the other hand, the six non-randomized or non-controlled trials, assessed using the ROBINS-I tool, emerged with a global moderate-level risk of bias (Figure 3). The first and sixth domains (bias due to confounding, bias in measurement of outcomes) resulted moderately in all five studies included. Moreover, two studies showed a moderate level of bias in the seventh domain (bias in selection of the reported result), and one study was evaluated with a moderate level of risk of bias in the fifth domain (bias due to missing data).

### 3.2. Sample Demographics

A total of 600 subjects were included from all the studies discussed. The mean age of the sample was 41.61 (2.13) years. A total of 51.1% of the sample was male, whereas 48.9% of subjects were female. Of the total subjects, 515 (85.8%) had a diagnosis of MDD, while 85 (14.2%) were affected by TRD. In most cases, moderate-to-severe depression was diagnosed. See Table 1 for more detailed information concerning each study.

### 3.3. Effects of a Single Dose of Psilocybin on Depressive Symptoms

Goodwin et al. [64] conducted a double-blind dose-finding parallel-group randomized trial, consisting of three groups of subjects with MDD: the first group (*n* = 79, 56% female, mean age: 40.2 ± 12.2) received a 25 mg dose of psilocybin, the second group (*n* = 75, 55% female, mean age: 40.6 ± 12.8) received a 10 mg dose of psilocybin, and the third group (*n* = 79, 46% female, mean age: 38.7 ± 11.7), considered a placebo, was given a 1 mg dose of psilocybin. Three weeks after the intervention, a decrease from baseline on the MADRS scale of 12 points was observed in the first group (i.e., 25 mg), of 7.9 points in the second group (i.e., 10 mg), and of 5.4 points in the third group (i.e., 1 mg). Following treatment, the mean difference between the first and third groups was statistically significant (mean difference: −6.6, 95% CI [−10.2; −2.9], *p <* 0.001), whereas the mean difference between the second and third groups was not (mean difference: −2.5; 95% CI [−6.2; 1.2], *p* = 0.18) [64]. The same research group conducted a subsequent open-label fixed-dose exploratory trial on 19 patients with TRD currently using SSRIs augmented with a single 25 mg dose of psilocybin [63]. At 3 weeks after treatment, a decrease of 14.9 points (95% CI [−20.7; −9.2]) was detected on the MADRS. Furthermore, such improvement in patients’ depressive symptoms severity was apparent at 2 days after treatment and maintained throughout the 3-week follow-up [63]. Based on these findings, Raison et al. [67] applied a randomized placebo-controlled trial on 104 patients with MDD, in which the treatment group (*n* = 51, 47% female, mean age: 40.4 ± 11.7) received a single dose of 25 mg psilocybin followed by niacin, whereas the control group (*n* = 53, 53% female, mean age: 41.8 ± 11.7) received two subsequent 100 mg doses of niacin. Forty-three days after treatment, the mean difference between the two groups was 12.3 (*p* < 0.001) in favor of the psilocybin-treated group [67]. Conversely, Sloshower et al. [68] conducted a placebo-controlled within-subject fixed-order trial on 19 patients with a diagnosis of MDD undergoing an initial administration of a placebo dose (i.e., microcrystalline cellulose) followed by the dispensation of a 0.3 mg/kg dose of psilocybin, 4 weeks apart. In partial contrast to the previously discussed studies, here depressive symptoms severity significantly decreased following both placebo and psilocybin intervention, with no significant difference in the degree of change between the two conditions. However, antidepressant effect sizes were larger after psilocybin (*d* = 1.02–2.27) than after placebo (*d* = 0.65–0.99) [68]. Lastly, von Rotz et al. [69] employed a double-blind randomized trial comprising 52 participants with MDD subdivided into two independent groups: the experimental group (*n* = 26, 65% female, mean age: 37.6 ± 10.9) was treated with a 0.215 mg/kg dose of psilocybin, whereas the control group (*n* = 26, 62% female, mean age: 37.6 ± 10.9) was given a placebo dose. Fourteen days after the intervention, the psilocybin administration resulted in an absolute decrease in depressive symptom severity of 13 points from baseline (95% CI [−15.0; −1.3], *p* < 0.01, *d* = 0.97) in the MADRS scores and reached an absolute decrement of 13.2 points (95% CI [−13.4; −1.3], *p* < 0.05, *d* = 0.67) in the BDI scores. Also, the depression-related subscale of SCL-90-R showed a statistically significant mean difference between the active and control groups following the treatment (mean difference: −0.83, 95% CI [0.18; 1.01], *p* < 0.01, *d* = 0.83) [69]. Table 2 shows more detailed specifics of each above-mentioned clinical trial in which a single dose of psilocybin was administered.

### 3.4. Effects of a Two-Dose Psilocybin Administration on Depressive Symptoms

Carhart-Harris et al. [59] conducted an open-label feasibility trial on 12 subjects with moderate-to-severe TRD in which two oral doses of psilocybin (10 mg and 25 mg) were administered 7 days apart. At 1-week after treatment, psilocybin markedly reduced depressive symptoms severity compared to baseline (mean difference: −11.8, 95% CI [−14.35; −9.15], *g* = 3.1, *p* < 0.01). Such an improvement in patients’ clinical conditions remained significant even at 3-month follow-up (mean difference: −9.2, 95% CI [−12.71; −5.69], *g* = 2, *p* < 0.01) [59]. The same research group carried out an additional pre-post neuroimaging study on 19 subjects with a diagnosis of TRD, applying the same intervention modalities as outlined above and also collecting self-reported clinical outcomes [60]. They highlighted that the delivery of two doses of psilocybin successfully reduced patients’ depressive symptoms severity at 5 weeks after treatment compared to baseline (mean difference: −8, *t* = −6.3, *p* < 0.001) [60]. Carhart-Harris et al. [61] conducted a third open-label single-arm trial, recruiting 20 subjects with TRD who received two doses of psilocybin (10 mg and 25 mg), spaced 7 days apart. Here, measurements of depressive symptoms severity were taken at baseline, 1-week, 2 weeks, 3 weeks, and 5 weeks after treatment, as well as at 3-month and 6-month follow-up. QIDS scores showed a significant decrease in all timepoints compared to baseline with the maximum effect size at 5 weeks (*t* = −7.2, *p* < 0.001, *d* =2.3). Moreover, of the 19 patients who completed the follow-up, all showed a reduction in depression intensity already at 1-week, and for most of them this clinical improvement persisted until 3–5 weeks [61]. Lyons and Carhart-Harris [66] carried out a further open-label pilot study on 15 individuals with a diagnosis of TRD, undergoing two dosing sessions: an initial safety dose (i.e., 10 mg) and a subsequent treatment dose (i.e., 25 mg) 1 week later. Clinical patients showed a significant decrease in BDI scores at 1-week after the psilocybin treatment compared to baseline (*t* = 7.9, 95% CI [16.17; 28.23], *p* < 0.001, *g* = 1.9) [66]. Unlike the above-mentioned investigations, Carhart-Harris et al. [30] recruited 59 patients with moderate-to-severe MDD in a double-blind randomized controlled trial. Participants were assigned to two independent groups: the treatment group (*n* = 30, 30% female, mean age: 43.3 ± 9.7) was given two 25 mg doses of psilocybin 3 weeks apart, along with a daily placebo for 6 weeks, whereas the active control group (*n* = 29, 31% female, mean age: 41.2 ± 11.7) received two placebo doses, also 3 weeks apart, but with daily escitalopram. Six weeks following the intervention, the mean QIDS scores dropped from baseline by 8 points in the treatment group and by 6 points in the active control one, leading to a non-statistically significant between-group difference equal to 2 points (95% CI [−5.0; 0.9]) [30]. Another randomized controlled trial was carried out by Davis et al. [62], who collected data from 24 subjects with MDD. Here, participants were split into two groups: the treatment group (*n* = 13, 69% female, mean age: 43.6 ± 13.0) received immediate medication consisting of two psilocybin administrations of 20 mg and 30 mg, respectively, whereas the control group (*n* = 11, 64% female, mean age: 35.2 ± 9.9) remained on the waiting list. Psilocybin successfully ameliorated patients’ depressive symptoms severity with larger effect sizes at 5 weeks (*d* = 2.5, 95% CI [1.4; 3.5], *p* < 0.001) and 8 weeks (*d* = 2.6, 95% CI [1.5; 3.7], *p* < 0.001) after treatment. Conversely, the control group remained stable at both timepoints compared to baseline [62]. Lastly, Gukasyan et al. [65] conducted a follow-up study by adding to the original sample of Davis et al. [62] those clinical patients who were previously included in the waiting list (and who had now received the same treatment as the experimental group). Compared to baseline, subjects’ depressive symptoms severity was markedly decreased across the following timepoints: 1-week, 1-month, 3-months, 6-months, and 12-months after treatment, with larger effect sizes at 6-months (*d* = 2.6) and 12-months (*d* = 2.4). The mean difference between the baseline scores and the various timepoints was statistically significant (*F* = 34.9, *p* < 0.001, *η*^2^*_p_* = 0.61), but no significant effect of condition (i.e., immediate versus delayed treatment) or a time-by-condition interaction was detected [65]. Table 3 shows more detailed specifics of each above-mentioned clinical trial in which two doses of psilocybin were administered.

### 3.5. Single-Dose versus Two-Dose Psilocybin Administration on Depressive Symptoms

**Figure 4:**
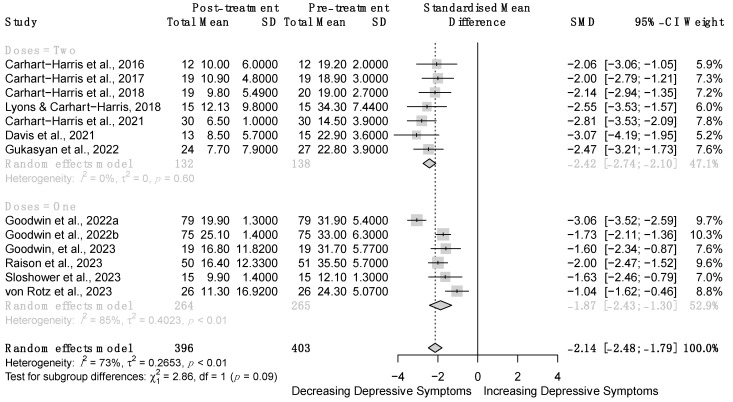
Forest plot of the meta-analysis conducted on pre- and post-treatment outcomes. SD = Standard Deviation, SMD = Standardized Mean Difference, 95% CI = 95% Confidence Interval [30,59,60,61,62,63,64,65,66,67,68,69].

**Figure 5:**
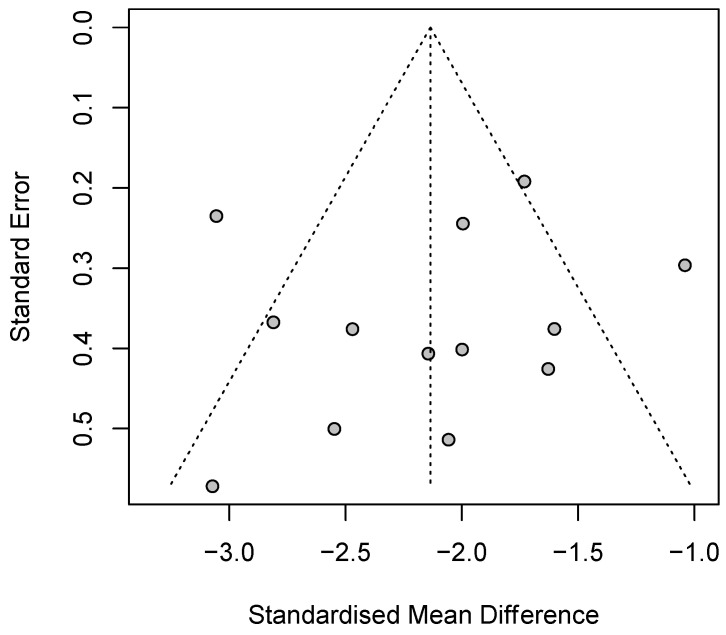
Funnel plot of the studies included in the meta-analysis on pre- and post-treatment outcomes.

Random-effect meta-analysis showed that psilocybin significantly reduces patients’ depressive symptoms severity compared to baseline (*z* = −12.11, *p* < 0.001, *d* = −2.14, 95% CI [−2.48; −1.78]; Figure 4). Such an effect was observed not only when single-dose psilocybin was administered (k = 6), but also when two-dose psilocybin was delivered (k = 7). Although two-dose psilocybin treatment displayed a more pronounced effect size compared to single-dose intervention (*d* = −2.42, 95% CI [−2.74; −2.11] versus *d* = −1.87, 95% CI [−2.43; −1.30]), the test for subgroups difference was not statistically significant (χ^2^_(1)_ = 2.86, *p* = 0.09). Overall heterogeneity resulting from effect size variability and sampling error was high (I^2^ = 73.1%, 95% CI [53.2%; 84.5%]; H = 1.93, 95% CI [1.46; 2.54], τ^2^ = 0.27, 95% CI [0.07; 0.86]), as well as the heterogeneity in the effect size distribution (Q_(12)_ = 44.62, *p* < 0.001). The funnel plot displayed minor signs of asymmetry and a few outliers (Figure 5). Nonetheless, such asymmetry was not statistically significant (*t*_(10)_ = −0.85, *p* = 0.39).

**Figure 6:**
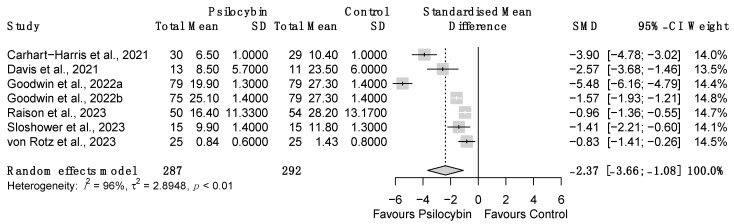
Forest plot of the meta-analysis conducted comparing the post-treatment outcomes among active psilocybin and control groups. SD = Standard Deviation, SMD = Standardized Mean Difference, 95% CI = 95% Confidence Interval [30,62,64,67,68,69].

The psilocybin effect on patients’ depressive symptoms severity was also compared to the consequences sorted by ‘inactive’ interventions (e.g., placebo, niacin), where control groups were included in the research designs. Random-effect meta-analysis showed that psilocybin significantly reduces subjects’ depressive symptoms severity compared to control (*z* = −3.60, *p* < 0.001, *d* = −2.37, 95% CI [−3.66; −1.08]; Figure 6). Subgroup analyses were not conducted in this case due to the low number of studies employing a control group/condition (k = 7). Overall heterogeneity resulting from effect size variability and sampling error was high (I^2^ = 96.3%, 95% CI [94.3%; 97.6%]; H = 5.23, 95% CI [4.20; 6.52], τ^2^ = 2.89, 95% CI [1.13; 14.39]), as well as the heterogeneity in the effect size distribution (Q_(6)_ = 164.37, *p* < 0.001).

## 4. Discussion

The aim of the present systematic review was firstly to summarize and analyze the current evidence concerning the therapeutic effects of a single- and a two-dose psilocybin administration with a specific focus on the treatment of TRD and MDD, including the latest trials, and then to compare the efficacy of these two different dosages on the severity of depressive symptoms reported by patients with a primary diagnosis of MDD or TRD. The twelve studies taken into consideration highlighted a pronounced efficacy of psilocybin for the treatment of MDD/TRD. Indeed, both single- and two-dose administration solutions proved their effectiveness in reducing depressive symptoms severity compared to baseline [30,61,62,63,64,65,67,68,69]. Moreover, studies involving a control group showed greater efficacy of psilocybin treatment compared to active or standard placebo [64,67,69], as well as compared to other pharmacological interventions, e.g., SSRIs (escitalopram; [30]). In both single- and double-dose approaches, therapeutic effects of psilocybin treatment were maintained over the long term, with decreases in standardized self-report measurements lasting up to 12 months after treatment in some cases [65]. Overall, these results are in line with the findings of previous systematic reviews and meta-analyses demonstrating the efficacy of psilocybin in ameliorating MDD/TRD symptoms severity [70,71,72]. However, in contrast to prior investigations highlighting that higher doses and two sessions of psilocybin treatment were associated with superior antidepressant effects [45,73,74], we did not observe a statistically significant difference in post-treatment depressive symptoms severity between single- and two-dose psilocybin administration. Indeed, such a presumed enhanced effect of multiple treatment sessions might stem from methodological factors other than the two-dose regimen itself, e.g., the variety of preparatory and integrative psychological sessions provided to patients before/after treatment and the dosage administered per session. Nevertheless, psilocybin shows a large effect size comparable to that of other cutting-edge approaches currently used for the treatment of MDD/TRD, including transcranial magnetic stimulation and ketamine [70,75,76,77,78]. Yet, the number of studies and the sample size available to date on the topic of the present work are still too small to allow a reliable comparison between these interventions [79]. Therefore, additional primary research on psilocybin efficacy on depressive disorders is needed to fill this gap.

Another relevant issue that still has to be addressed regards the risk-benefit profile associated with both single- and two-dose administration [80]. Among the included studies, psilocybin was well tolerated by the vast majority of the patients undergoing both single- and two-dose regimens, and rare serious or unexpected adverse events occurred. Drug-related side effects were especially reported by participants within the first day after treatment, encompassing headache, nausea, dizziness, fatigue, palpitations, confusion, and a transient increase in their blood pressure [30,59,62,63,64,65,67,69]. Severe adverse events were only observed in a small portion of patients who reported suicidal ideation, intentional self-injury (i.e., nonsuicidal self-injurious behavior), and suicidal behavior [64], symptoms that might also be linked to MDD/TRD. Considering the similarity between the effect sizes of the two treatment approaches, a feasible strategy to maintain the therapeutic effect and minimize the risk of incurring these unwanted drug-related consequences might be to opt for the single-dose regimen. Nonetheless, forthcoming investigations could focus on comparing the safety profiles associated with single- and two-dose psilocybin administration in order to clarify the potential risks and benefits of different dosing regimens. A related key point to be further explored could be whether the enhanced outcomes associated with the two-dose intervention detected by previous studies sufficiently justify the inherent risks of a dual psilocybin administration [45]. Beyond the risk itself, the requirement for doubled psychological support and a double pharmacological dose could make the psilocybin-assisted treatment less affordable to those in need. A better understanding of these matters would significantly enhance the generalizability and appeal of psilocybin usage within clinical settings.

The present systematic review and meta-analysis suffers, however, from major limitations. First, most of the clinical trials included in the review had a small sample size (i.e., under 25 participants), which inevitably affects the external validity of this work. Moreover, the percentage of women included in the original studies was unexpectedly low. Although such a population appears to be two to three times more likely to receive a formal diagnosis of MDD [81,82], in half of the selected papers (i.e., 6 out of 12), the percentage of female participants was 50% or less. Second, almost half of the studies were open-label and were assessed by the authors with a moderate risk of bias, highlighting the lack of randomized controlled trials concerning the research topic within the current literature. Third, treatment protocols appeared markedly heterogeneous across the studies, complicating a direct comparison between the trials. Fourth, several impacting factors were left out of the meta-analysis, e.g., illness duration, eventual comorbidities, and number of previous treatments, therefore making our results limitedly reliable and in need of corroboration by future studies.

Further investigations and reviews are necessary to address not only the efficacy of psilocybin on depressive symptoms severity but also the effects of such a treatment on higher-order cognitive functions. A possible candidate to be tested could be delay discounting, i.e., the depreciation of the value of a reward related to the delay to its receipt [83,84,85], as it has been shown to be steeper among patients with MDD [86], and amenable to modulation by non-invasive treatment approaches [87]. Furthermore, subjects with MDD show lower confidence in their judgements and dysfunctional (meta-)cognitive abilities, such as decentering, metacognitive beliefs, and aspects of self-conscious attention [88]. These metacognitive domains, which can also be modulated by non-invasive brain stimulation [89], might be affected by psilocybin as well through the induction of synaptic plasticity mechanisms [90,91].

## 5. Conclusions

The results from the clinical trials analyzed in this systematic review and meta-analysis underline the efficacy of psilocybin in the treatment of MDD patients, identifying this psychedelic tryptamine as a possible tool for the treatment of such disorders. Indeed, comparing the effects of a single and two-dose psilocybin administration, both treatment strategies were associated with a significant decrease in depressive symptoms severity compared to baseline assessment. Nonetheless, subsequent clinical trials, systematic reviews, and meta-analyses aimed at furthering this research topic are essential. With the results provided by future investigations, it could be possible to elaborate a more in-depth analysis of the available efficacy data, thus favoring the construction of evidence-based guidelines for intervention that would make the use of psilocybin within clinical settings more feasible and reliable.

## Figures and Tables

**Table 1:** Summary of the demographic characteristics of the samples from all the included studies.

ID	Sample Size (*n*)	% Female	Age, Mean (DS)	Diagnosis with Severity
Carhart-Harris et al., 2016 [59]	12	50%	42.6 (10.2)	TRD: moderate-severe, HAMD = 19.2
Carhart-Harris et al., 2017 [60]	19	21%	42.8 (10.1)	TRD: severe, QIDS = 18.9
Carhart-Harris et al., 2018 [61]	20	30%	44.1 (11)	TRD: severe, BDI = 34.5
Lyons and Carhart-Harris, 2018 [66]	15	27%	45.4 (2.9)	TRD: moderate-severe, BDI = 34.3
Carhart-Harris et al., 2021 [30]	59	34%.	41.2	MDD: moderate-severe, BDI = 29.1
Davis et al., 2021 [62]	24	67%	39.8 (12.2)	MDD: moderate-severe, HAMD = 22.8
Goodwin et al., 2022 [64]	233	52%	39.8 (12.2)	MDD: moderate (30%), Severe (68%)
Gukasyan et al., 2022 [65]	24	67%	39.8 (12.2)	MDD: moderate-severe, HAMD = 22.8
Goodwin et al., 2023 [63]	19	58%	42.2 (10.8)	TRD: moderate; MADRS = 31.7
Raison et al., 2023 [67]	104	50%	41.1 (11.3)	MDD: moderate-severe, MADRS = 35.25
Sloshower et al., 2023 [68]	19	68%	42.8 (13.8)	MDD: moderate-severe, HAMD = 22.57
von Rotz et al., 2023 [69]	52	63%.	36.75	MDD: moderate, BDI = 27.35

Notes: MDD = Major Depressive Disorder, TRD = Treatment-resistant Depression, BDI = Beck Depression Inventory, HAMD = Hamilton Rating Scale for Depression, MADRS = Montgomery-Åsberg Depression Rating Scale, QIDS = Quick Inventory of Depressive Symptomatology, DS = standard deviation.

**Table 2:** Summary of the characteristics of the studies in which a single-dose psilocybin was used.

ID	Study Design	Treatments	Psilocybin Dosage	Control (Dosage)	Clinical Measures	Effects
Goodwin et al. (2022) [64]	RCT	Three preparatory meetings, one dose of psilocybin with music, and two psychological integration sessions	10 mg or 25 mg	Psilocybin (1 mg)	MADRS	Mean MADRS scores decreased by 12 points in the first group (25 mg), by 7.9 points in the second group (10 mg), and by 5.4 in the third group (1 mg) at 3 weeks after treatment
Goodwin et al. (2023) [63]	Open-label	Three preparatory meetings, one dose of psilocybin with music, and two integration sessions	25 mg	-	MADRS, QIDS	Mean MADRS scores decreased by 14.9 points at week 3 weeks after treatment
Raison et al. (2023) [67]	RCT	Preparatory sessions totaling 6–8 h, one dose of psilocybin or niacin with music, followed by 4 h integration sessions	25 mg	Niacin (100 mg)	MADRS	Significant reduction in mean MADRS scores in the treatment group (−19.1) compared to controls (−6.8) from baseline to 43 days after treatment
Sloshower et al. (2023) [68]	Placebo-controlledwithin-subjects	2 h preparatory sessions, a single dose of psilocybin or placebo, and two sessions of psychotherapy	0.3 mg/kg	Placebo	HAMD, QIDS	Greater reduction in mean QIDS scores following psilocybin administration (∆ = 6.3–8.7) than placebo (∆ = 4.4–5.8) at 2 weeks after treatment
von Rotz et al. (2023) [69]	RCT	Two preparatory sessions, one dose of psilocybin or placebo, and three integration sessions	0.215 mg/kg	Placebo	MADRS, BDI	Reduction in mean MADRS scores in the treatment group (−13.2) compared to baseline, with an effect size significantly larger than placebo at 2 weeks after treatment

Notes: RCT: Randomized Controlled Trial, BDI = Beck Depression Inventory, HAMD = Hamilton Depression Rating Scale, MADRS = Montgomery-Åsberg Depression Rating Scale, QIDS = Quick Inventory of Depressive Symptomatology.

**Table 3:** Summary of the characteristics of the studies in which two doses of psilocybin were used.

ID	Study Design	Treatments	First Psilocybin Dosage	Second Psilocybin Dosage	Control (Dosage)	Clinical Measures	Effects
Carhart-Harris et al. (2016) [59]	Open-label	Preparatory sessions totaling 4 h, two doses of psilocybin with music, 1-week apart, and two integration sessions	10 mg	25 mg	-	QIDS, BDI, HAMD, MADRS	Decrease in QIDS scores compared to baseline (−11.8) at 1-week after treatment. A further decrement in mean QIDS scores (−9.2) was observed at 3-month follow-up
Carhart-Harris et al. (2017) [60]	Open-label	Preparatory sessions totaling 4 h, two doses of psilocybin with music, 1-week apart, and two integration sessions	10 mg	25 mg	-	QIDS	Reduced QIDS scores compared to baseline (−8.0) at 5 weeks after treatment
Carhart-Harris et al. (2018) [61]	Open-label	Preparatory sessions totaling 4 h, two doses of psilocybin with music, 1-week apart. After each dose, an integrative session was conducted	10 mg	25 mg	-	QIDS, BDI, HAMD	Reduction in mean QIDS scores compared to baseline at 1-week, 2 weeks, 3 weeks, 5 weeks, and 3 months after treatment. BDI scores were significantly lower compared to baseline at 1-week, 3-months, and 6-months after treatment
Lyons and Carhart-Harris (2018) [66]	Open-label	Preparatory sessions totaling 4 h, two doses of psilocybin with music, 1-week apart, and two integration sessions	10 mg	25 mg	-	BDI, HAMD	Decrease in BDI scores (−22.2) compared to baseline at 1-week after treatment
Carhart-Harris et al. (2021) [30]	RCT	Preparatory sessions totaling 3 h, two doses of psilocybin or escitalopram, 3 weeks apart, and an integrative session	25 mg	25 mg	Psilocybin (1 mg)	QIDS, BDI, HAMD, MADRS	At 6 weeks, the mean QIDS change score compared to baseline was −6.0 in the escitalopram group and −8.0 in the psilocybin group
Davis et al. (2021) [62]	Randomized waiting-list	Preparatory sessions totaling 8 h, two doses of psilocybin with music, spaced ~2 weeks apart, and two integration sessions	20 mg	30 mg	Waiting list	HAMD, QIDS	Mean HAMD scores decreased in the immediate treatment group compared to baseline at 1-week (−8.0) and 1-month (8.5) after treatment. Mean HAMD scores remained stable in subjects belonging to the waiting list
Gukasyan et al. (2022) [65]	Follow-up	Preparatory sessions totaling 8 h, two doses of psilocybin with music, spaced ~2 weeks apart, and two integration sessions	20 mg	30 mg	-	HAMD, QIDS	Mean HAMD scores decreased in both immediate and delayed treatment groups compared to baseline at 1-week, 1-month, 3-months, 6-months, and 12-months after treatment

Notes: RCT: Randomized Controlled Trial, BDI = Beck Depression Inventory, HAMD = Hamilton Depression Rating Scale, MADRS = Montgomery-Åsberg Depression Rating Scale, QIDS = Quick Inventory of Depressive Symptomatology.

## Data Availability

Scripts and datasets used for conducting the meta-analysis are available by contacting the corresponding author under proper request.

## References

[1] Bromet E., Andrade L.H., Hwang I., Sampson N.A., Alonso J., de Girolamo G., de Graaf R., Demyttenaere K., Hu C., Iwata N. (2011). Cross-National Epidemiology of DSM-IV Major Depressive Episode. BMC Med..

[2] Ferrari A.J., Somerville A.J., Baxter A.J., Norman R., Patten S.B., Vos T., Whiteford H.A. (2013). Global Variation in the Prevalence and Incidence of Major Depressive Disorder: A Systematic Review of the Epidemiological Literature. Psychol. Med..

[3] American Psychiatric Association (2022). Diagnostic and Statistical Manual of Mental Disorders: DSM-5-TR.

[4] GBD 2019 Mental Disorders Collaborators (2022). Global, Regional, and National Burden of 12 Mental Disorders in 204 Countries and Territories, 1990–2019: A Systematic Analysis for the Global Burden of Disease Study 2019. Lancet Psychiatry.

[5] Greenberg P.E., Fournier A.-A., Sisitsky T., Simes M., Berman R., Koenigsberg S.H., Kessler R.C. (2021). The Economic Burden of Adults with Major Depressive Disorder in the United States (2010 and 2018). PharmacoEconomics.

[6] Karrouri R., Hammani Z., Benjelloun R., Otheman Y. (2021). Major Depressive Disorder: Validated Treatments and Future Challenges. World J. Clin. Cases.

[7] Fava M., Davidson K.G. (1996). Definition and Epidemiology of Treatment-Resistant Depression. Psychiatr. Clin. N. Am..

[8] Gaynes B.N., Lux L., Gartlehner G., Asher G., Forman-Hoffman V., Green J., Boland E., Weber R.P., Randolph C., Bann C. (2020). Defining Treatment-Resistant Depression. Depress. Anxiety.

[9] Dodd S., Bauer M., Carvalho A.F., Eyre H., Fava M., Kasper S., Kennedy S.H., Khoo J.-P., Lopez Jaramillo C., Malhi G.S. (2021). A Clinical Approach to Treatment Resistance in Depressed Patients: What to Do When the Usual Treatments Don’t Work Well Enough?. World J. Biol. Psychiatry Off. J. World Fed. Soc. Biol. Psychiatry.

[10] Monteiro W.O., Noshirvani H.F., Marks I.M., Lelliott P.T. (1987). Anorgasmia from Clomipramine in Obsessive-Compulsive Disorder. A Controlled Trial. Br. J. Psychiatry J. Ment. Sci..

[11] Montejo A.I., Llorca G., Izquierdo J.A., Ledesma A., Bousoño M., Calcedo A., Carrasco J.L., Daniel E., de Dios A., de la Gándara J. (1996). Sexual dysfunction secondary to SSRIs. A comparative analysis in 308 patients. Actas Luso. Esp. Neurol. Psiquiatr. Cienc. Afines.

[12] Goldstein B.J., Goodnick P.J. (1998). Selective Serotonin Reuptake Inhibitors in the Treatment of Affective Disorders—III. Tolerability, Safety and Pharmacoeconomics. J. Psychopharmacol..

[13] De Wilde J., Spiers R., Mertens C., Bartholomé F., Schotte G., Leyman S. (1993). A Double-Blind, Comparative, Multicentre Study Comparing Paroxetine with Fluoxetine in Depressed Patients. Acta Psychiatr. Scand..

[14] Sussman N., Ginsberg D. (1998). Rethinking Side Effects of the Selective Serotonin Reuptake Inhibitors: Sexual Dysfunction and Weight Gain. Psychiatr. Ann..

[15] Haddad P. (1998). The SSRI Discontinuation Syndrome. J. Psychopharmacol..

[16] Culpepper L. (2001). Early Onset of Antidepressant Action: Impact on Primary Care. J. Clin. Psychiatry.

[17] Greden J.F. (2002). Unmet Need: What Justifies the Search for a New Antidepressant?. J. Clin. Psychiatry.

[18] Fergusson D., Doucette S., Glass K.C., Shapiro S., Healy D., Hebert P., Hutton B. (2005). Association between Suicide Attempts and Selective Serotonin Reuptake Inhibitors: Systematic Review of Randomised Controlled Trials. BMJ.

[19] Gunnell D., Saperia J., Ashby D. (2005). Selective Serotonin Reuptake Inhibitors (SSRIs) and Suicide in Adults: Meta-Analysis of Drug Company Data from Placebo Controlled, Randomised Controlled Trials Submitted to the MHRA’s Safety Review. BMJ.

[20] Hammad T.A., Laughren T.P., Racoosin J.A. (2006). Suicide Rates in Short-Term Randomized Controlled Trials of Newer Antidepressants. J. Clin. Psychopharmacol..

[21] Khan A., Warner H.A., Brown W.A. (2000). Symptom Reduction and Suicide Risk in Patients Treated with Placebo in Antidepressant Clinical Trials: An Analysis of the Food and Drug Administration Database. Arch. Gen. Psychiatry.

[22] Khan A., Khan S.R., Leventhal R.M., Brown W.A. (2001). Symptom Reduction and Suicide Risk in Patients Treated with Placebo in Antidepressant Clinical Trials: A Replication Analysis of the Food and Drug Administration Database. Int. J. Neuropsychopharmacol..

[23] Khan A., Khan S., Kolts R., Brown W.A. (2003). Suicide Rates in Clinical Trials of SSRIs, Other Antidepressants, and Placebo: Analysis of FDA Reports. Am. J. Psychiatry.

[24] Ling S., Ceban F., Lui L.M.W., Lee Y., Teopiz K.M., Rodrigues N.B., Lipsitz O., Gill H., Subramaniapillai M., Mansur R.B. (2022). Molecular Mechanisms of Psilocybin and Implications for the Treatment of Depression. CNS Drugs.

[25] Geiger H.A., Wurst M.G., Daniels R.N. (2018). DARK Classics in Chemical Neuroscience: Psilocybin. ACS Chem. Neurosci..

[26] Vollenweider F.X., Vollenweider-Scherpenhuyzen M.F., Bäbler A., Vogel H., Hell D. (1998). Psilocybin Induces Schizophrenia-like Psychosis in Humans via a Serotonin-2 Agonist Action. Neuroreport.

[27] Moliner R., Girych M., Brunello C.A., Kovaleva V., Biojone C., Enkavi G., Antenucci L., Kot E.F., Goncharuk S.A., Kaurinkoski K. (2023). Psychedelics Promote Plasticity by Directly Binding to BDNF Receptor TrkB. Nat. Neurosci..

[28] Lamanna J., Isotti F., Ferro M., Spadini S., Racchetti G., Musazzi L., Malgaroli A. (2022). Occlusion of Dopamine-Dependent Synaptic Plasticity in the Prefrontal Cortex Mediates the Expression of Depressive-like Behavior and Is Modulated by Ketamine. Sci. Rep..

[29] Krystal J.H., Kavalali E.T., Monteggia L.M. (2024). Ketamine and Rapid Antidepressant Action: New Treatments and Novel Synaptic Signaling Mechanisms. Neuropsychopharmacology.

[30] Carhart-Harris R., Giribaldi B., Watts R., Baker-Jones M., Murphy-Beiner A., Murphy R., Martell J., Blemings A., Erritzoe D., Nutt D.J. (2021). Trial of Psilocybin versus Escitalopram for Depression. N. Engl. J. Med..

[31] Nikolin S., Rodgers A., Schwaab A., Bahji A., Zarate C.J., Vazquez G., Loo C. (2023). Ketamine for the Treatment of Major Depression: A Systematic Review and Meta-Analysis. EClinicalMedicine.

[32] Yavi M., Lee H., Henter I.D., Park L.T., Zarate C.A.J. (2022). Ketamine Treatment for Depression: A Review. Discov. Ment. Health.

[33] Psiuk D., Nowak E.M., Dycha N., Łopuszańska U., Kurzepa J., Samardakiewicz M. (2022). Esketamine and Psilocybin-The Comparison of Two Mind-Altering Agents in Depression Treatment: Systematic Review. Int. J. Mol. Sci..

[34] Kopra E.I., Ferris J.A., Winstock A.R., Young A.H., Rucker J.J. (2022). Adverse Experiences Resulting in Emergency Medical Treatment Seeking Following the Use of Magic Mushrooms. J. Psychopharmacol..

[35] Yerubandi A., Thomas J.E., Bhuiya N.M.M.A., Harrington C., Villa Zapata L., Caballero J. (2024). Acute Adverse Effects of Therapeutic Doses of Psilocybin: A Systematic Review and Meta-Analysis. JAMA Netw. Open.

[36] Johnson M.W., Griffiths R.R., Hendricks P.S., Henningfield J.E. (2018). The Abuse Potential of Medical Psilocybin According to the 8 Factors of the Controlled Substances Act. Neuropharmacology.

[37] van Amsterdam J., Opperhuizen A., van den Brink W. (2011). Harm Potential of Magic Mushroom Use: A Review. Regul. Toxicol. Pharmacol. RTP.

[38] De la Fuente Revenga M., Jaster A.M., McGinn J., Silva G., Saha S., González-Maeso J. (2022). Tolerance and Cross-Tolerance among Psychedelic and Nonpsychedelic 5-HT(2A) Receptor Agonists in Mice. ACS Chem. Neurosci..

[39] Brown R.T., Nicholas C.R., Cozzi N.V., Gassman M.C., Cooper K.M., Muller D., Thomas C.D., Hetzel S.J., Henriquez K.M., Ribaudo A.S. (2017). Pharmacokinetics of Escalating Doses of Oral Psilocybin in Healthy Adults. Clin. Pharmacokinet..

[40] Husain M.I., Blumberger D.M., Castle D.J., Ledwos N., Fellows E., Jones B.D.M., Ortiz A., Kloiber S., Wang W., Rosenblat J.D. (2023). Psilocybin for Treatment-Resistant Depression without Psychedelic Effects: Study Protocol for a 4-Week, Double-Blind, Proof-of-Concept Randomised Controlled Trial. BJPsych Open.

[41] Rucker J., Jafari H., Mantingh T., Bird C., Modlin N.L., Knight G., Reinholdt F., Day C., Carter B., Young A. (2021). Psilocybin-Assisted Therapy for the Treatment of Resistant Major Depressive Disorder (PsiDeR): Protocol for a Randomised, Placebo-Controlled Feasibility Trial. BMJ Open.

[42] Dodd S., Norman T.R., Eyre H.A., Stahl S.M., Phillips A., Carvalho A.F., Berk M. (2022). Psilocybin in Neuropsychiatry: A Review of Its Pharmacology, Safety, and Efficacy. CNS Spectr..

[43] Breeksema J.J., Niemeijer A., Krediet E., Karsten T., Kamphuis J., Vermetten E., van den Brink W., Schoevers R. (2024). Patient Perspectives and Experiences with Psilocybin Treatment for Treatment-Resistant Depression: A Qualitative Study. Sci. Rep..

[44] Johnson M., Richards W., Griffiths R. (2008). Human Hallucinogen Research: Guidelines for Safety. J. Psychopharmacol..

[45] Yu C.-L., Liang C.-S., Yang F.-C., Tu Y.-K., Hsu C.-W., Carvalho A.F., Stubbs B., Thompson T., Tsai C.-K., Yeh T.-C. (2022). Trajectory of Antidepressant Effects after Single- or Two-Dose Administration of Psilocybin: A Systematic Review and Multivariate Meta-Analysis. J. Clin. Med..

[46] Page M.J., McKenzie J.E., Bossuyt P.M., Boutron I., Hoffmann T.C., Mulrow C.D., Shamseer L., Tetzlaff J.M., Akl E.A., Brennan S.E. (2021). The PRISMA 2020 Statement: An Updated Guideline for Reporting Systematic Reviews. Int. J. Surg..

[47] Methley A.M., Campbell S., Chew-Graham C., McNally R., Cheraghi-Sohi S. (2014). PICO, PICOS and SPIDER: A Comparison Study of Specificity and Sensitivity in Three Search Tools for Qualitative Systematic Reviews. BMC Health Serv. Res..

[48] Cates C.J., Cheng H.-Y., Corbett M.S., Eldridge S.M., Sterne J.A.C., Savović J., Page M.J., Elbers R.G., Blencowe N.S., Boutron I. (2019). RoB 2: A Revised Tool for Assessing Risk of Bias in Randomised Trials. BMJ.

[49] Sterne J.A., Hernán M.A., Reeves B.C., Savović J., Berkman N.D., Viswanathan M., Henry D., Altman D.G., Ansari M.T., Boutron I. (2016). ROBINS-I: A Tool for Assessing Risk of Bias in Non-Randomised Studies of Interventions. BMJ.

[50] Higgins J.P.T., Thompson S.G., Deeks J.J., Altman D.G. (2003). Measuring Inconsistency in Meta-Analyses. BMJ.

[51] Egger M., Davey Smith G., Schneider M., Minder C. (1997). Bias in Meta-Analysis Detected by a Simple, Graphical Test. BMJ.

[52] Agrawal M., Richards W., Beaussant Y., Shnayder S., Ameli R., Roddy K., Stevens N., Richards B., Schor N., Honstein H. (2024). Psilocybin-Assisted Group Therapy in Patients with Cancer Diagnosed with a Major Depressive Disorder. Cancer.

[53] Griffiths R.R., Johnson M.W., Carducci M.A., Umbricht A., Richards W.A., Richards B.D., Cosimano M.P., Klinedinst M.A. (2016). Psilocybin Produces Substantial and Sustained Decreases in Depression and Anxiety in Patients with Life-Threatening Cancer: A Randomized Double-Blind Trial. J. Psychopharmacol..

[54] Ross S., Bossis A., Guss J., Agin-Liebes G., Malone T., Cohen B., Mennenga S.E., Belser A., Kalliontzi K., Babb J. (2016). Rapid and Sustained Symptom Reduction Following Psilocybin Treatment for Anxiety and Depression in Patients with Life-Threatening Cancer: A Randomized Controlled Trial. J. Psychopharmacol..

[55] Barba T., Buehler S., Kettner H., Radu C., Cunha B.G., Nutt D.J., Erritzoe D., Roseman L., Carhart-Harris R. (2022). Effects of Psilocybin versus Escitalopram on Rumination and Thought Suppression in Depression. BJPsych Open.

[56] Doss M.K., Považan M., Rosenberg M.D., Sepeda N.D., Davis A.K., Finan P.H., Smith G.S., Pekar J.J., Barker P.B., Griffiths R.R. (2021). Psilocybin Therapy Increases Cognitive and Neural Flexibility in Patients with Major Depressive Disorder. Transl. Psychiatry.

[57] Goodwin G.M., Aaronson S.T., Alvarez O., Atli M., Bennett J.C., Croal M., DeBattista C., Dunlop B.W., Feifel D., Hellerstein D.J. (2023). Single-Dose Psilocybin for a Treatment-Resistant Episode of Major Depression: Impact on Patient-Reported Depression Severity, Anxiety, Function, and Quality of Life. J. Affect. Disord..

[58] Roseman L., Nutt D.J., Carhart-Harris R.L. (2017). Quality of Acute Psychedelic Experience Predicts Therapeutic Efficacy of Psilocybin for Treatment-Resistant Depression. Front. Pharmacol..

[59] Carhart-Harris R.L., Bolstridge M., Rucker J., Day C.M.J., Erritzoe D., Kaelen M., Bloomfield M., Rickard J.A., Forbes B., Feilding A. (2016). Psilocybin with Psychological Support for Treatment-Resistant Depression: An Open-Label Feasibility Study. Lancet Psychiatry.

[60] Carhart-Harris R.L., Roseman L., Bolstridge M., Demetriou L., Pannekoek J.N., Wall M.B., Tanner M., Kaelen M., McGonigle J., Murphy K. (2017). Psilocybin for Treatment-Resistant Depression: fMRI-Measured Brain Mechanisms. Sci. Rep..

[61] Carhart-Harris R.L., Bolstridge M., Day C.M.J., Rucker J., Watts R., Erritzoe D.E., Kaelen M., Giribaldi B., Bloomfield M., Pilling S. (2018). Psilocybin with Psychological Support for Treatment-Resistant Depression: Six-Month Follow-Up. Psychopharmacology.

[62] Davis A.K., Barrett F.S., May D.G., Cosimano M.P., Sepeda N.D., Johnson M.W., Finan P.H., Griffiths R.R. (2021). Effects of Psilocybin-Assisted Therapy on Major Depressive Disorder: A Randomized Clinical Trial. JAMA Psychiatry.

[63] Goodwin G.M., Croal M., Feifel D., Kelly J.R., Marwood L., Mistry S., O’Keane V., Peck S.K., Simmons H., Sisa C. (2023). Psilocybin for Treatment Resistant Depression in Patients Taking a Concomitant SSRI Medication. Neuropsychopharmacology.

[64] Goodwin G.M., Aaronson S.T., Alvarez O., Arden P.C., Baker A., Bennett J.C., Bird C., Blom R.E., Brennan C., Brusch D. (2022). Single-Dose Psilocybin for a Treatment-Resistant Episode of Major Depression. N. Engl. J. Med..

[65] Gukasyan N., Davis A.K., Barrett F.S., Cosimano M.P., Sepeda N.D., Johnson M.W., Griffiths R.R. (2022). Efficacy and Safety of Psilocybin-Assisted Treatment for Major Depressive Disorder: Prospective 12-Month Follow-Up. J. Psychopharmacol..

[66] Lyons T., Carhart-Harris R.L. (2018). More Realistic Forecasting of Future Life Events After Psilocybin for Treatment-Resistant Depression. Front. Psychol..

[67] Raison C.L., Sanacora G., Woolley J., Heinzerling K., Dunlop B.W., Brown R.T., Kakar R., Hassman M., Trivedi R.P., Robison R. (2023). Single-Dose Psilocybin Treatment for Major Depressive Disorder: A Randomized Clinical Trial. JAMA.

[68] Sloshower J., Skosnik P.D., Safi-Aghdam H., Pathania S., Syed S., Pittman B., D’Souza D.C. (2023). Psilocybin-Assisted Therapy for Major Depressive Disorder: An Exploratory Placebo-Controlled, Fixed-Order Trial. J. Psychopharmacol..

[69] Von Rotz R., Schindowski E.M., Jungwirth J., Schuldt A., Rieser N.M., Zahoranszky K., Seifritz E., Nowak A., Nowak P., Jäncke L. (2023). Single-Dose Psilocybin-Assisted Therapy in Major Depressive Disorder: A Placebo-Controlled, Double-Blind, Randomised Clinical Trial. EClinicalMedicine.

[70] Fang S., Yang X., Zhang W. (2024). Efficacy and Acceptability of Psilocybin for Primary or Secondary Depression: A Systematic Review and Meta-Analysis of Randomized Controlled Trials. Front. Psychiatry.

[71] Haikazian S., Chen-Li D.C.J., Johnson D.E., Fancy F., Levinta A., Husain M.I., Mansur R.B., McIntyre R.S., Rosenblat J.D. (2023). Psilocybin-Assisted Therapy for Depression: A Systematic Review and Meta-Analysis. Psychiatry Res..

[72] Metaxa A.-M., Clarke M. (2024). Efficacy of Psilocybin for Treating Symptoms of Depression: Systematic Review and Meta-Analysis. BMJ.

[73] Li N.-X., Hu Y.-R., Chen W.-N., Zhang B. (2022). Dose Effect of Psilocybin on Primary and Secondary Depression: A Preliminary Systematic Review and Meta-Analysis. J. Affect. Disord..

[74] Perez N., Langlest F., Mallet L., De Pieri M., Sentissi O., Thorens G., Seragnoli F., Zullino D., Kirschner M., Kaiser S. (2023). Psilocybin-Assisted Therapy for Depression: A Systematic Review and Dose-Response Meta-Analysis of Human Studies. Eur. Neuropsychopharmacol..

[75] Wei Y., Zhu J., Pan S., Su H., Li H., Wang J. (2017). Meta-Analysis of the Efficacy and Safety of Repetitive Transcranial Magnetic Stimulation (rTMS) in the Treatment of Depression. Shanghai Arch. Psychiatry.

[76] Brini S., Brudasca N.I., Hodkinson A., Kaluzinska K., Wach A., Storman D., Prokop-Dorner A., Jemioło P., Bala M.M. (2023). Efficacy and Safety of Transcranial Magnetic Stimulation for Treating Major Depressive Disorder: An Umbrella Review and Re-Analysis of Published Meta-Analyses of Randomised Controlled Trials. Clin. Psychol. Rev..

[77] Alnefeesi Y., Chen-Li D., Krane E., Jawad M.Y., Rodrigues N.B., Ceban F., Di Vincenzo J.D., Meshkat S., Ho R.C.M., Gill H. (2022). Real-World Effectiveness of Ketamine in Treatment-Resistant Depression: A Systematic Review & Meta-Analysis. J. Psychiatr. Res..

[78] Saccenti D., Lodi L., Moro A.S., Scaini S., Forresi B., Lamanna J., Ferro M. (2024). Novel Approaches for the Treatment of Post-Traumatic Stress Disorder: A Systematic Review of Non-Invasive Brain Stimulation Interventions and Insights from Clinical Trials. Brain Sci..

[79] Button K.S., Ioannidis J.P.A., Mokrysz C., Nosek B.A., Flint J., Robinson E.S.J., Munafò M.R. (2013). Power Failure: Why Small Sample Size Undermines the Reliability of Neuroscience. Nat. Rev. Neurosci..

[80] Bender D., Hellerstein D.J. (2022). Assessing the Risk–Benefit Profile of Classical Psychedelics: A Clinical Review of Second-Wave Psychedelic Research. Psychopharmacology.

[81] Otte C., Gold S.M., Penninx B.W., Pariante C.M., Etkin A., Fava M., Mohr D.C., Schatzberg A.F. (2016). Major Depressive Disorder. Nat. Rev. Dis. Primers.

[82] Bangasser D.A., Valentino R.J. (2014). Sex Differences in Stress-Related Psychiatric Disorders: Neurobiological Perspectives. Front. Neuroendocrinol..

[83] Moro A.S., Saccenti D., Vergallito A., Gregori Grgič R., Grazioli S., Pretti N., Crespi S., Malgaroli A., Scaini S., Ruggiero G.M. (2024). Evaluating the Efficacy of Transcranial Magnetic Stimulation in Symptom Relief and Cognitive Function in Obsessive–Compulsive Disorder, Substance Use Disorder, and Depression: An Insight from a Naturalistic Observational Study. Appl. Sci..

[84] Moro A.S., Saccenti D., Ferro M., Scaini S., Malgaroli A., Lamanna J. (2023). Neural Correlates of Delay Discounting in the Light of Brain Imaging and Non-Invasive Brain Stimulation: What We Know and What Is Missed. Brain Sci..

[85] Moro A.S., Saccenti D., Seccia A., Ferro M., Malgaroli A., Lamanna J. (2023). Poke And Delayed Drink Intertemporal Choice Task (POKE-ADDICT): An Open-Source Behavioral Apparatus for Intertemporal Choice Testing in Rodents. Anim. Models Exp. Med..

[86] Amlung M., Marsden E., Holshausen K., Morris V., Patel H., Vedelago L., Naish K.R., Reed D.D., McCabe R.E. (2019). Delay Discounting as a Transdiagnostic Process in Psychiatric Disorders: A Meta-Analysis. JAMA Psychiatry.

[87] Moro A.S., Saccenti D., Vergallito A., Scaini S., Malgaroli A., Ferro M., Lamanna J. (2023). Transcranial Direct Current Stimulation (tDCS) over the Orbitofrontal Cortex Reduces Delay Discounting. Front. Behav. Neurosci..

[88] Drueke B., Gauggel S., Weise L., Forkmann T., Mainz V. (2022). Metacognitive Judgements and Abilities in Patients with Affective Disorders. Curr. Psychol..

[89] Saccenti D., Moro A.S., Sassaroli S., Malgaroli A., Ferro M., Lamanna J. (2024). Neural Correlates of Metacognition: Disentangling the Brain Circuits Underlying Prospective and Retrospective Second-Order Judgments through Noninvasive Brain Stimulation. J. Neurosci. Res..

[90] Mograbi D.C., Rodrigues R., Bienemann B., Huntley J. (2024). Brain Networks, Neurotransmitters and Psychedelics: Towards a Neurochemistry of Self-Awareness. Curr. Neurol. Neurosci. Rep..

[91] Ferro M., Lamanna J., Spadini S., Nespoli A., Sulpizio S., Malgaroli A. (2022). Synaptic Plasticity Mechanisms behind TMS Efficacy: Insights from Its Application to Animal Models. J. Neural Transm..

